# Loss of ADAM29 does not affect viability and fertility in mice but improves wound healing

**DOI:** 10.1016/j.isci.2024.110135

**Published:** 2024-05-29

**Authors:** Diana Campos-Iglesias, Alejandro A. Montero, Francisco Rodríguez, Carlos López-Otín, José M.P. Freije

**Affiliations:** 1 Departamento de Bioquímica y Biología Molecular, Instituto Universitario de Oncología del Principado de Asturias (IUOPA), Universidad de Oviedo, Oviedo, Spain; 2 Instituto de Investigación Sanitaria del Principado de Asturias (ISPA), Oviedo, Spain; 3 Unidad de Transgénicos, Bioterio e Imagen Preclínica, Servicios Científico-Técnicos, Instituto Universitario de Oncología del Principado de Asturias (IUOPA), Universidad de Oviedo, Oviedo, Spain; 4 Facultad de Ciencias de la Vida y la Naturaleza, Universidad Nebrija, Madrid, Spain; 5 Centre de Recherche des Cordeliers, Université de Paris Cité, Sorbonne Université, INSERM U1138, Paris, France; 6 Centro de Investigación Biomédica en Red de Cáncer (CIBERONC), Madrid, Spain

**Keywords:** Traumatology, Molecular biology

## Abstract

ADAM29 (a disintegrin and metalloprotease domain 29) is a member of the membrane-anchored ADAM family of proteins, which is highly expressed in testis and may mediate different physiological and pathological processes. Although the functions of many ADAM family members have been well characterized, the biological relevance of ADAM29 has remained largely unknown. Here, we report the generation of an *Adam29*-deficient mouse model to delve deeper into the *in vivo* functions of this ADAM family member. We show that ADAM29 depletion does not affect mice viability, development, or fertility, but somehow impinges on metabolism and energy expenditure. We also report herein that ADAM29 deficiency leads to an accelerated wound healing process, without affecting cell reprogramming in mouse-derived fibroblasts. Collectively, our findings provide new insights into ADAM29 biological functions, highlighting the importance of non-catalytic ADAM proteases.

## Highlights

• A novel *Adam29* knock-out mouse model was generated using CRISPR-Cas9 technology
• ADAM29 is not essential for development, growth, or fertility
• ADAM29 deficiency affects energy balance in mice
• Deficiency of ADAM29 accelerates wound healing from the initial stages of the process
• ADAM29 does not behave as a cell reprogramming barrier in mouse-derived fibroblasts

## Graphical abstract

**Figure:**
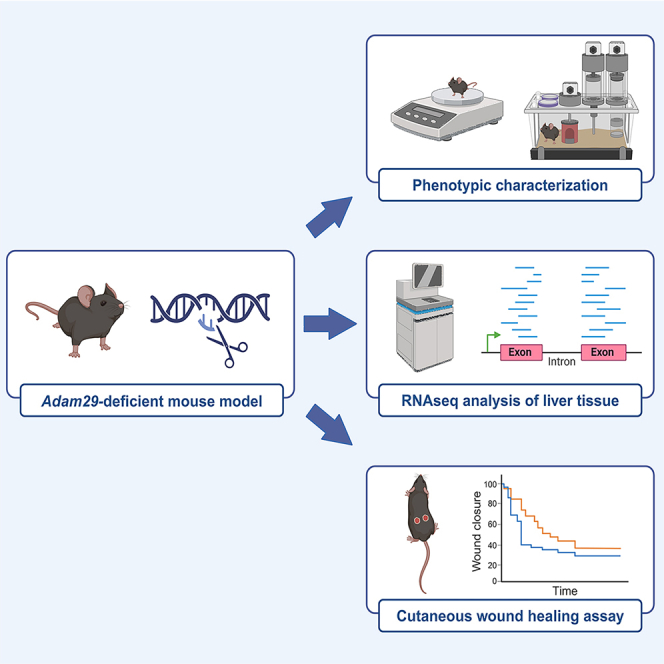

## Introduction

The ADAM (a disintegrin and metalloproteinase) proteins are membrane-anchored and secreted enzymes that play important roles in different physiological processes, such as fertilization, development and differentiation, immune responses, and regenerative activities.^1^^,^^2^ Upon extracellular stress conditions, ADAMs can trigger rapid cellular responses through the modulation of intra- and intercellular signaling cascades. Thus, unsurprisingly, dysregulation of multiple proteins of this family has been linked to different pathologies, including various human cancers, inflammatory and autoimmune diseases, cardiovascular alterations, and neurological disorders.^3^^,^^4^ ADAMs are type I transmembrane proteins that share a common multidomain structure: a prodomain, a metalloproteinase domain, a disintegrin domain, a cysteine-rich domain, an EGF-like (or membrane-proximal) domain, a transmembrane domain, and a cytoplasmatic domain.^5^ In humans, 22 members of the ADAM family have been described but, strikingly, eight of them (ADAM2, 7, 11, 18, 22, 23, 29, and 32) lack one or more residues of the consensus motif HExGHxxGxxHD required for catalysis, therefore being proteolytically inactive.^6^ However, these non-active ADAM family members may function as scaffolding proteins or cell-cell/cell-matrix signaling regulators.^7^ Considering that each domain has distinct functions and that the presence of the disintegrin domain in ADAMs is unique among cell-surface proteins, it has been reported that these proteases can modulate cell-matrix and cell-cell interactions by binding to integrins.^8^^,^^9^ Thus, the functional exploration of the less-studied, non-catalytic ADAMs has become of particular interest. In this regard, sequence alterations of the non-catalytic ADAM29 have been described in human melanoma, affecting the adhesion of melanoma cells *in vitro.*^10^ Moreover, both *ADAM29* mutations and changes in its expression levels were related to other types of human cancer, such as esophageal, gastric, colorectal, renal, and breast cancers.^11^^,^^12^^,^^13^^,^^14^^,^^15^^,^^16^^,^^17^ Interestingly, it has also been reported that the LPL/ADAM29 expression ratio could be a prognosis indicator in chronic lymphocytic leukemia.^18^^,^^19^

In this work, we aimed to delve deeper into the biological functions of ADAM29. Using CRISPR-Cas9 technology, we generated an *Adam29-*deficient mouse model. These mice are viable and fertile, with no obvious abnormalities or deviations from the Mendelian ratios in their offspring. However, we noticed some alterations in the metabolism of mice lacking ADAM29, which could be related to a different fuel selection as the main energy source in these animals. Finally, we have shown that *Adam29* ablation in mice leads to an accelerated wound healing process. Altogether, these data point to new biological functions for ADAM29.

## Results

### ADAM29 is not essential for growth, development, and fertility of adult mice but impinges on metabolism

**Figure 1:**
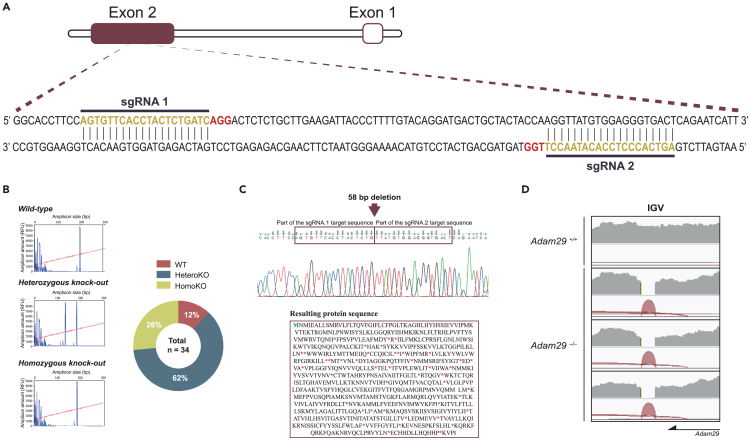
CRISPR-Cas9 mediated generation of the new *Adam29* knock-out mouse model (A) Representation of the *Adam29* locus with the position of exons and target sequences for the two sgRNAs (dark yellow). The PAM sequence in each case is depicted in red. (B) Representative examples of capillary-electrophoresis-based fragment analysis of the resulting pups after Cas9:sgRNA microinjection. The par-to-whole graph at right shows the percentage of wild-type (WT), heterozygous knock-out (HeteroKO), and homozygous knock-out (HomoKO) animals born (total pups = 34). (C) Nucleotide (upper panel) and amino acid (lower panel) sequences of the homozygous knock-out mice selected as founders. Red boxes represent partial sequence target for both sgRNAs. The initial methionine is marked in green. Premature stop codons are indicated with red asterisks. (D) Integrative Genomics Viewer (IGV)-inspected sequence reads of RNA-seq data from *Adam29* locus in *Adam29*^*+/+*^ and *Adam29*^*−/−*^ mice.

To evaluate the role of ADAM29 *in vivo*, we generated a mouse model deficient for this metalloprotease by using a CRISPR-Cas9-based technology. For this purpose, we designed two different sgRNAs targeting the only coding exon of the *Adam29* gene (Figure 1A). Using a fragment analysis technique, we could discern three different genotypes: *wild-type* animals, with no Cas9 cleavage, and two subtypes of *knock-out* animals, homozygous and heterozygous *knock-out*, with exactly the same or different deletions in both alleles, respectively (Figure 1B). To confirm *Adam29* interruption, we performed Sanger sequencing of all the animals. We then selected as founders those carrying the same deletion of 58 pb, causing the disruption of the *Adam29* reading frame and, in consequence, the appearance of various premature stop codons (Figure 1C). As ADAM29 is highly expressed in testis, we next examined RNA sequencing (RNA-seq) data from *Adam29*^*+/+*^ and *Adam29*^*−/−*^ testis, using the Integrative Genome Viewer (IGV). As shown in Figure 1D, there were no reads mapped between both sgRNA target sites, confirming the 58 bp deletion and, subsequently, *Adam29* disruption in knockout mice.

**Figure 2:**
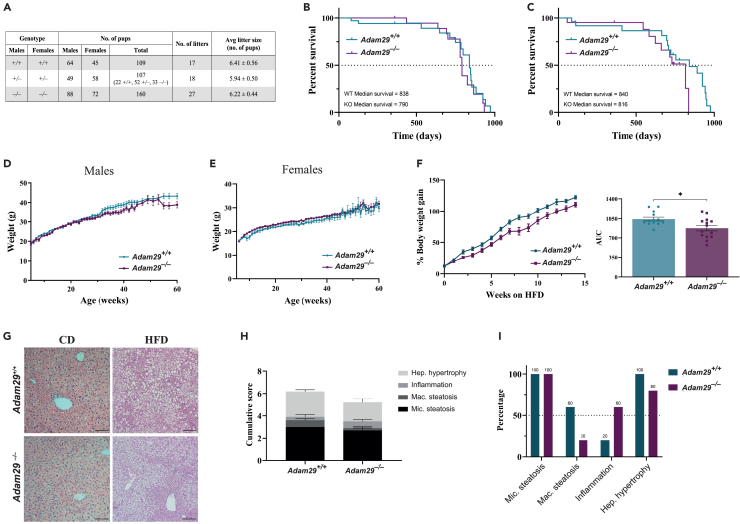
*Adam29* is not essential for growth, development and fertility of adult mice (A) Offspring from multiple matings between *Adam29*^*+/+*^, *Adam29*^*+/−*^, and *Adam29*^*−/−*^ mice. (B and C) Kaplan-Meier survival plots of *Adam29*^*+/+*^ and *Adam29*^*−/−*^ male (B, *n* = 34 and *n* = 22, respectively) and female (C, *n* = 24 and *n* = 21, respectively) mice. (D and E) Body weight curves of *Adam29*^*+/+*^ and *Adam29*^*−/−*^ male (D, *n* = 34 and *n* = 25, respectively) and female (E, *n* = 10 and *n* = 27, respectively) mice kept on a standard chow diet. Data are mean ± SEM. (F) Cumulative body weight gain of *Adam29*^*+/+*^ (*n* = 13) and *Adam29*^*−/−*^ (*n* = 14) male mice kept on a high-fat diet (HFD) and analysis of both AUC (area under the curve, arbitrary units). Data are mean ± SEM, ∗*p* < 0.05, two-tailed Student’s t test. (G) Representative microphotographs of H&E stained liver sections of chow diet and high-fat diet fed *Adam29*^*+/+*^ mice. Scale bar: 100 μm. (H) Cumulative score of NAFLD of *Adam29*^*+/+*^ (*n* = 10) and *Adam29*^*−/−*^ (*n* = 10) male mice kept on an HFD. Data are mean ± SEM. (I) Incidence of different parameters scored in NAFLD analysis. Specific percentage values are indicated above the bars. Hep. hypertrophy, hepatocyte hypertrophy; Mac. steatosis, macrovacuolar steatosis; mic. steatosis, microvacuolar steatosis.

Considering that *Adam29* is highly expressed in testis, we decided to analyze the offspring from multiple matings, in order to investigate whether this metalloproteinase is essential for male fertility (Figure 2A). Heterozygous mice for the disrupted gene were interbred to generate *Adam29*^*+/+*^, *Adam29*^*+/−*^, and *Adam29*^*−/−*^ mice. Out of 107 pups born, 22 were *Adam*^*+/+*^, 52 were *Adam29*^*+/−*^, and 33 were *Adam29*^*−/−*^, close to the expected Mendelian ratios 1:2:1. To further determine if the fertility of *Adam29*^*−/−*^ males was affected, we carried out multiple matings between *Adam29*^*+/+*^ and *Adam29*^*−/−*^ mice separately. We found no significant difference in the average number of pups per litter (6.41 ± 0.56 for *Adam29*^*+/+*^, 5.94 ± 0.50 for *Adam29*^*+/−*^, and 6.22 ± 0.44 for *Adam29*^*−/−*^ mice). These results suggest that *Adam29*-deficiency does not affect male or female fertility in mice.

Additionally, *Adam29*-deficient mice were viable without any obvious alteration. Thus, we did not find any significant difference in survival rates between *Adam29*^*+/+*^ and *Adam29*^*−/−*^mice, neither in males (Figure 2B) nor in females (Figure 2C). Comparison of body weight evolution under standard chow diet showed a similar rate of body weight gain between both genotypes (Figures 2D and 2E), although we noticed a slight decrease in body weight of *Adam29*^*−/−*^ male mice. When fed a high fat diet (HFD), *Adam29*^*−/−*^ mice increased their body weight at a lower rate compared with their *Adam29*^*+/+*^ littermates (Figures 2F and 2G). However, no significant differences between genotypes were observed when weighting liver, spleen, or gonadal white adipose tissue (Figures S1A–S1C), nor in glucose homeostasis (Figures S1D–S1I) or in plasma levels of adiponectin and leptin (Figures S1J and S1K). Furthermore, there were no significant differences between *Adam29*^*+/+*^ and *Adam29*^*−/−*^ mice in plasma biochemical parameters, including glucose, cholesterol, and other parameters associated with liver, kidney, and pancreatic function (Table 1). However, when conducting histological analysis of liver sections (Figure S2), we observed that ADAM29-deficient mice exhibited lower histological scores for non-alcoholic liver disease (NAFLD) (Figure 2H). Although 100% of both *Adam29*^*+/+*^ and *Adam29*^*−/−*^ mice presented microvacuolar steatosis, the incidence of macrovacuolar steatosis and hepatocyte hypertrophy was reduced in *Adam29*^*−/−*^ animals, whereas inflammation incidence was higher in these animals (Figure 2I). These results suggest a potentially different liver response to a high-fat diet and fat accumulation within the liver of *Adam29*^*−/−*^ mice.

**Table 1:** Plasma biochemical parameters in Adam29^+/+^ and Adam29^−/−^ HFD-fed mice

Parameters	*Adam29*^*+/+*^	*Adam29*^*−/−*^
Glucose (mg/dL)	387.6 ± 21.53	393.7 ± 24.39
Total cholesterol (mg/dL)	181.5 ± 7.57	187.2 ± 5.48
Total protein (g/dL)	4.76 ± 0.11	5.13 ± 0.18
ALP (U/L)	82.82 ± 2.81	88.92 ± 3.64
Albumin (g/dL)	3.21 ± 0.09	3.35 ± 0.07
ALT (U/L)	73.80 ± 5.31	79.00 ± 8.68
Globulin (g/dL)	1.63 ± 0.07	1.79 ± 0.20
A/G Ratio	1.95 ± 0.09	1.59 ± 0.09^a^
BUN (mg/dL)	18.68 ± 0.52	17.41 ± 0.53
Creatinin (mg/dL)	0.31 ± 0.01	0.32 ± 0.02
B/C Ratio	60.25 ± 2.46	54.25 ± 2.04
Urea (mg/dL)	39.96 ± 1.11	37.18 ± 1.25
Amylase (U/L)	349.1 ± 15.99	373.2 ± 18.33
Lipase (U/L)	41.67 ± 0.62	43.89 ± 2.04
Calcium (mg/dL)	8.66 ± 0.19	8.93 ± 0.19
Phosphorus (mg/dL)	9.54 ± 0.76	7.47 ± 0.53^a^

ALP, alkaline phosphatase; ALT, alanine aminotransferase; A/G, albumin/creatinine; BUN, blood urea nitrogen; B/C, blood urea nitrogen/creatinine. *Adam29*^*+/+*^*n* = 11, *Adam29*^*−/−*^ mice *n* = 12.

a *p* < 0.05, two-tailed Student’s t test.

**Figure 3:**
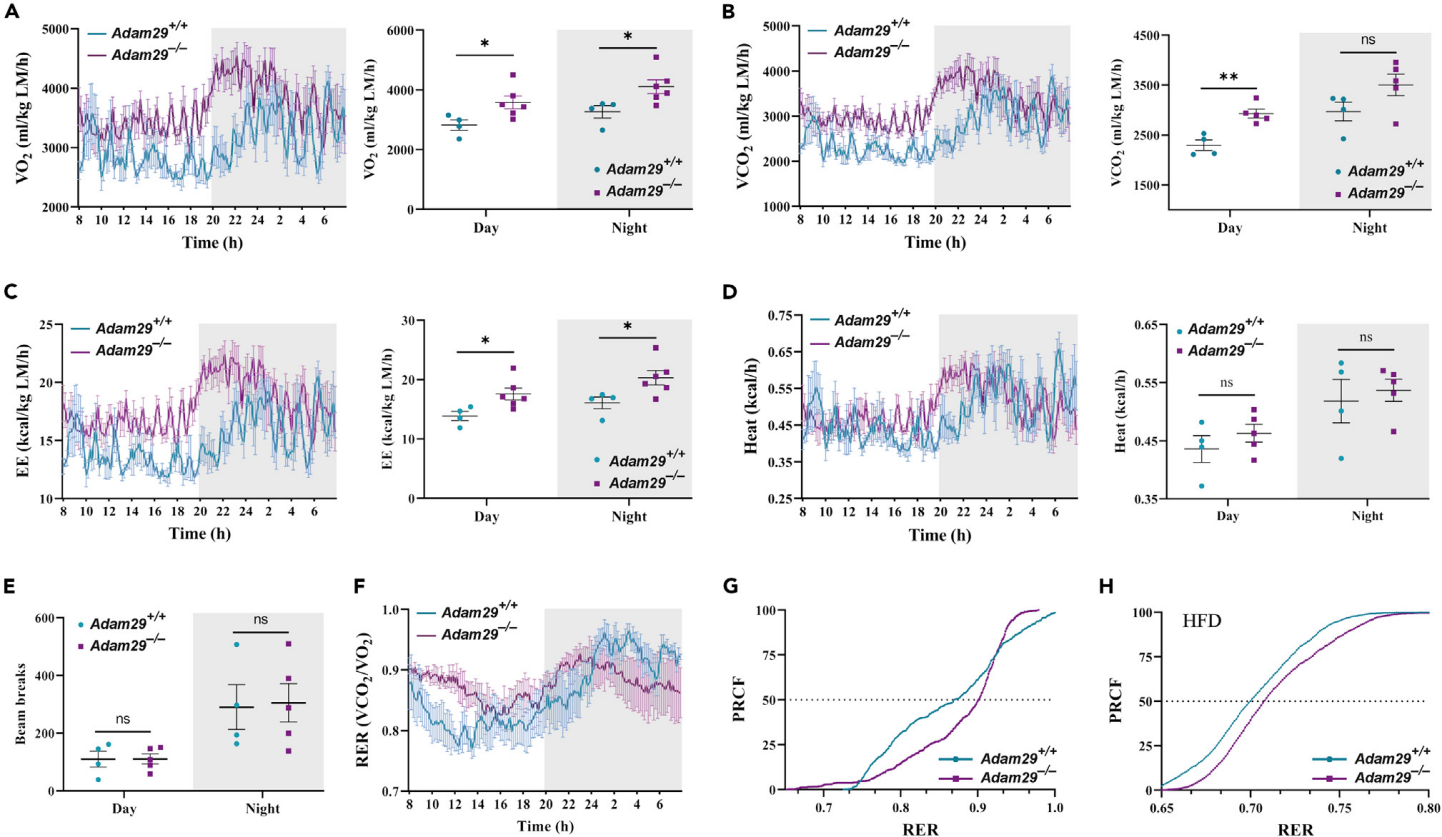
ADAM29-deficient mice show an altered metabolism and an increased metabolic flexibility (A–F) Indirect calorimetry measured with the Oxymax-CLAMS system in 5–6-month-old males (*Adam29*^*+/+*^*n* = 4, *Adam29*^*−/−*^*n* = 5). White and gray background indicate daytime and nighttime analysis, respectively. (A) Oxygen consumption (VO_2,_ weight normalized), (B) carbon dioxide production (VCO_2_, weight normalized), (C) energy expenditure (EE, weight normalized), and (D) heat production during a 24-h period are represented on each left panel. Data presented on each right panel are mean ± SEM, ∗*p* < 0.05, ∗∗*p* < 0.01, ns, non-significant, two-tailed Student’s t test. *Adam29*^*+/+*^ VO_2_ and EE night datasets did not pass the normality test (*p* values of 0.0430 and 0.0494, respectively). LM, lean mass. (E) Total ambulatory activity of *Adam29*^*+/+*^ and *Adam29*^*−/−*^ mice measured by the number of beam breaks. (F) Respiratory exchange ratio (RER) during light and dark cycles. (G and H) Percent relative cumulative frequency (PRCF) curves generated from RER values of *Adam29*^*+/+*^ and *Adam29*^*−/−*^ males fed with standard chow diet (CD) or 9–10 weeks of high-fat diet (HFD), respectively (CD-fed *Adam29*^*+/+*^*n* = 4, *Adam29*^*−/−*^*n* = 5, HFD-fed *Adam29*^*+/+*^*n* = 13, *Adam29*^*−/−*^*n* = 14).

To further investigate whether ADAM29 deficiency could impinge on energy expenditure, we subjected *Adam29*^*+/+*^ and *Adam29*^*−/−*^ male mice to indirect calorimetry using Oxymax CLAMS. Both oxygen consumption (VO_2_) and carbon dioxide production (VCO_2_) of *Adam29*^*−/−*^ mice were elevated (Figures 3A and 3B), also showing an elevated energy expenditure when compared with control littermates (Figure 3C). There were no significant differences in heat production (Figure 3D), neither in locomotor activity (Figure 3E). However, we noticed that the respiratory exchange ratio (RER) tended to be slightly increased in ADAM29 deficient mice, especially during daytime (Figure 3F). This could suggest that *Adam29*^*−/−*^ mice may favor carbohydrates as a fuel source. To evaluate possible differences in the capacity to adapt fuel selection between carbohydrates and fatty acids as the main energy source, known as metabolic flexibility, we analyzed the percent relative cumulative frequency (PRCF) curves of RER values^20^ between *Adam29*^*+/+*^ and *Adam29*^*−/−*^ mice. Interestingly, ADAM29 deficient mice had a rightward shift in the RER distribution, even when kept on an HFD, which could indicate a higher metabolic flexibility toward carbohydrate oxidation (Figures 3G and 3H). Collectively, these results demonstrate that ADAM29 deficiency is compatible with normal mouse development and fertility, but somehow impinges on energy balance. In this regard, further studies will be needed to unveil the specific role of ADAM29 in the regulation of metabolism.

### RNA-seq analysis reveals different gene expression in ADAM29 deficient mice

**Figure 4:**
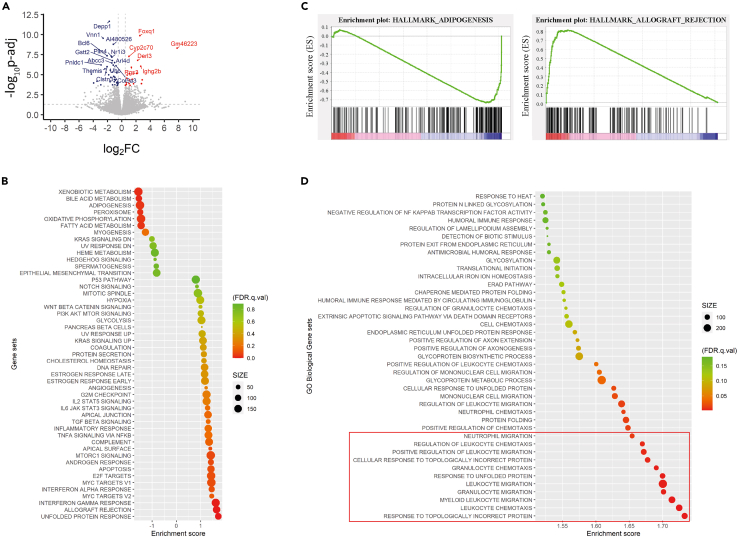
RNA-seq analysis reveals changes in gene expression between *Adam29*^*+/+*^ and *Adam29*^*−/−*^ mice (A) Volcano plot showing -log_10_ (adjusted *p* value) versus log_2_ fold change (log_2_FC) of differentially expressed genes between *Adam29*^*+/+*^ and *Adam29*^*−/−*^ liver samples. Each dot represents a single gene. Red and blue dotes denote significant upregulated and downregulated genes, respectively (adjusted *p* value < 0.05). The 20 most altered genes are named. (B) GSEA bubble plot. The vertical axis represents the gene set names used for the analysis (MH: mouse hallmark gene sets), and the horizontal axis represents the enrichment score (NES). The number of genes included in each pathway is expressed by the size of each point. (C) Enrichment plots for Adipogenesis and Allograft_Rejection gene sets enriched in GSEA Hallmark analysis, showing the profile of the enrichment score (ES) and the positions of gene set members on the rank-ordered list. (D) GSEA bubble plot using GO Biological Processes as the gene sets. Only positively regulated gene sets (NES > 1) and those whose family-wise error rate (FWER) is <1 are shown in the graph. The vertical axis represents the gene set names and the horizontal axis the enrichment score (NES). The number of genes included in each pathway is expressed by the size of each point. The red box remarks those gene sets that show NES over 1.65.

To better understand how ADAM29 contributed to the observed phenotype, we performed RNA-seq gene expression analysis using liver samples from *Adam29*^*+/+*^ and *Adam29*^*−/−*^ mice (Table S1). We found several differentially expressed genes (DEGs) between both genotypes (Figure S3). In this sense, only those showing an adjusted *p* value <0.05 and log_2_ fold-change >0.5 or <−0.5 were considered DEGs for further analysis. Using these criteria, we identified 62 DEGs, being 22 of them upregulated and 40 downregulated in *Adam29*^*−/−*^ mice (Figure 4A). To gain a better understanding of the observed differences, we performed gene set enrichment analysis (GSEA) from the molecular signature database (MSigDB) using hallmark and Gene Ontology Biological Process gene sets. In this sense, GSEA analysis of hallmark gene sets (Figures 4B and 4C; Table S2A) revealed an enrichment of downregulated genes related to bile acid metabolism, oxidative phosphorylation and fatty acid metabolism in ADAM29 deficient mice, which reinforces the role of ADAM29 in metabolism. Conversely, we found an upregulation of allograft rejection and interferon-response gene sets in *Adam29*^*−/−*^ mice, suggesting that ADAM29 may be involved in some immune activities. Interestingly, when performing GSEA analysis of Gene Ontology Biological Process we observed an enrichment in *Adam29*^*−/−*^ mice of genes involved in the migration of different cell types (Figure 4D; Table S2B), which suggests that ADAM29 may have an influence on biological processes dependent on cell migration. Finally, as endoplasmic reticulum stress has been implicated in liver injury such as nonalcoholic steatohepatitis (NASH), we analyzed *Xbp1* unprocessed and spliced transcript levels. However, we did not observe any remarkable difference between *Adam29*^*+/+*^ and *Adam29*^*−/−*^ unchallenged mice (Table S3).

### ADAM29 deficiency accelerates wound healing in mice but does not affect cell reprogramming

**Figure 5:**
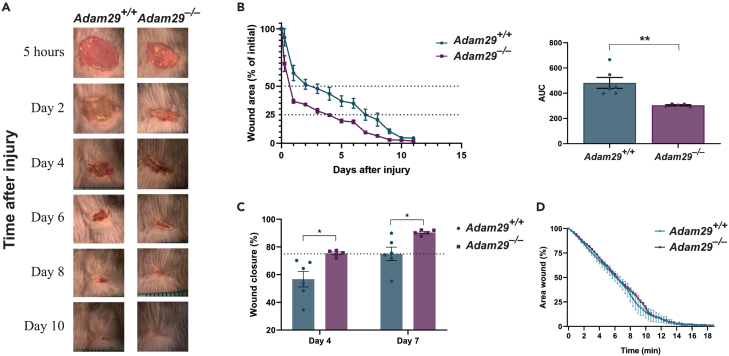
Cutaneous wound healing is accelerated in ADAM29 deficient mice Full thickness excisional wounds were generated on the back skin of *Adam29*^*+/+*^ (*n* = 6) and *Adam29*^*−/−*^ (*n* = 5) animals, and wound closure percentage was monitored over 11 days. (A) Representative photographs of skin wounds showing macroscopic wound closure on different time points post-injury. (B) Wound area closure rates from *Adam29*^*+/+*^ and *Adam29*^*−/−*^ mice were determined at each point. The rates are presented as percentage of the initial wound area (day 0). The right panel in (B) shows a quantitative measurement of area under the curve (AUC). Data are represented as mean ± SEM, two-tailed Student’s t test with Welch’s correction, ∗∗*p* < 0.01. (C) Percentage of wound closure from *Adam29*^*+/+*^ and *Adam29*^*−/−*^ mice at day 4 and day 7 post-injury. The dashed line indicates 75% of wound closure percentage. Data are represented as mean ± SEM, two-tailed Student’s t test with Welch’s correction, ∗*p* < 0.05. (D) Quantification of the wounded closure area expressed as the remaining area uncovered by the cells in *Adam29*^*+/+*^ and *Adam29*^*−/−*^ MEFs. Three biological replicates of each genotype were used to perform the experiment. Data are represented as mean ± SEM.

To shed more light on the role of ADAM29 in biological processes where cell migration is key, we studied the cutaneous wound healing process in ADAM29-deficient mice. For this purpose, we performed 7-mm full-thickness excisional wounds on the back skin of 8-10-week-old *Adam29*^*+/+*^ and *Adam29*^*−/−*^ animals. The wound closure was then monitored over the following 11 days, by measuring the wound area percentage versus the initial area (Figures 5A and 5B). We found that the healing process was accelerated in *Adam29*^*−/−*^ mice, where 75% of wound closure was achieved on day 4 post-incision (24.61 ± 0.96% of the initial wound remained open at day 4), while getting that percentage of closure took 7 days in *Adam29*^*+/+*^ control mice (24.97 ± 4.79% of the initial wound area remained open at day 7) (Figure 5C). This significant acceleration observed in ADAM29 deficient mice was already noticeable as early as 5 h post-injury, when *Adam29*^*−/−*^ animals showed a wound closure of 30.33 ± 6.81%, in contrast to the 7.34 ± 7.56% achieved by *Adam29*^*+/+*^ controls. These results highlight how ADAM29 clearly influences wound healing since the very first steps of the process. As cellular behavior could mediate wound healing processes, we next performed an *in vitro* scratch assay to explore whether ADAM29 loss could affect fibroblast migration. However, as shown in Figure 5D, we did not observe any significant differences in scratch closure between *Adam29*^*+/+*^ and *Adam29*^*−/−*^ mouse embryonic fibroblasts (MEFs).

Finally, considering that ADAM29 was pointed out as a reprogramming barrier in human fibroblasts,^21^ we wondered whether murine ADAM29 could also be important in this process, impinging on cellular plasticity. To achieve this goal, we reprogrammed 12 different established MEF cell lines into induced pluripotent stem cells (iPSCs) through retroviral transduction of the pluripotency factors OCT4, SOX2, and KLF4, and 20 days post-infection, we stained the resulting colonies, without observing any significant difference between both genotypes (Figure S4). These results suggest that, contrary to the results described in human cells, *Adam29*-deficiency does not facilitate reprogramming of murine fibroblasts.

## Discussion

In this work, we have generated mice deficient in ADAM29 to evaluate the potential roles of this non-catalytic protease in homeostasis maintenance. Based on established roles for different ADAMs in fertility,^22^ and considering that ADAM29 seems to be preferentially expressed in testis, it seemed reasonable to hypothesize that ADAM29 may also contributed to this process. Nevertheless, we could not identify any nonredundant role for ADAM29 in fertility or development in mice. Interestingly, although some testis-specifically expressed ADAMs (such as ADAM1A, ADAM2, ADAM3, ADAM6, ADAM7, and ADAM24) are essential for male fertility,^23^^,^^24^^,^^25^^,^^26^^,^^27^^,^^28^ there are others including ADAM21 and ADAM32,^29^^,^^30^ which seem to be dispensable for reproduction in mice, as we have shown herein for ADAM29. This functional redundancy could protect spermatogenesis from gene loss of function and may also provide alternative ways to produce sperm under different stressful conditions. However, during phenotypic characterization, we noticed some alterations in the metabolism of mice lacking ADAM29, which suggests that this protease might be important in this context. Interestingly, ADAM19 and ADAM28 have also been found to modulate weight and glucose metabolism in mice.^31^^,^^32^

Of note, the disintegrin-domain of some of the catalytically inactive human ADAMs, including ADAM29, was shown to support integrin α4-dependent cell adhesion.^9^ Integrins are transmembrane regulators that dictate different cellular responses based on their interactions with extracellular matrix components or other transmembrane proteins in the neighboring cells. Thus, they can modulate cell adhesion, survival, and proliferation, as well as different cellular metabolic pathways in a reciprocal manner.^33^ Here, we have shown that energy metabolism seems to be regulated differently in *Adam29*-deficient mice, possibly impinging on fat accumulation pattern within the liver and weight gain. Remarkably, deficiency of integrin α4 in mice alleviates obesity-associated metabolic dysregulation, modulating the retention of macrophages in obese adipose tissue.^34^ Furthermore, knock-in mice expressing loss-of-function mutant integrin α4 were also protected from the development of obesity-induced insulin resistance, showing a dramatic reduction of monocyte/macrophage migration to adipose tissue.^35^

Given the critical role of integrins in the re-epithelization process,^36^ we have evaluated the relevance of ADAM29 in physiological wound healing. Our findings revealed that the ablation of *Adam29* in mice leads to an accelerated wound healing process. Notably, the difference between both genotypes was already observable a few hours post-injury, suggesting that ADAM29 role is specifically related to the initial stages of this process. Again, several integrins are essential for wound migration, as it is exemplified in keratinocyte-specific knock-out mice of β1-integrins, which show a severe retardation in wound re-epithelization.^37^ Interestingly, ADAM12 was found elevated in chronic skin wounds, and its absence in mice markedly accelerated keratinocyte migration.^38^ Furthermore, *Adam9* knockout mice also exhibit an accelerated wound healing and, interestingly, it has been shown that this protease binds α3β1-integrin on human keratinocyte cell lines, inducing cell migration.^39^^,^^40^ Of note, recruitment and migration of different immune cells, including monocytes/macrophages, is essential for tissue restoration following damage.^41^ Thus, we hypothesize that ADAM29 may be involved in the regulation of macrophage functions, maybe mediated by its interaction with integrin α4. However, further studies will be needed in this context. Finally, we explored whether ADAM29 could affect reprogramming in mouse-derived fibroblasts, as this protein was previously identified as a human cell reprogramming barrier.^21^ However, we did not observe any significant difference in iPSCs colony formation between *Adam29*^*−/−*^ and *Adam29*^*+/+*^ cells. Considering that some specific parts of the reprogramming process are unique to humans or mice,^42^ we hypothesize that ADAM29 may not be essential for reprogramming in mice. Still, a deeper evaluation of ADAM29 role on both human and mouse cell reprogramming is needed to clarify its functions.

In summary, the generation of *Adam29*-deficient mice has allowed us to identify novel roles of this protease in metabolism and wound healing processes. Moreover, we have demonstrated that mice lacking *Adam29* have normal fertility. These results confirm the importance of non-catalytic ADAM proteases, being *Adam29*-deficient mice a valuable experimental model to study novel functions of this protease *in vivo*.

### Limitations of the study

In our study, we generated an *Adam29*-deficient mouse model to delve deeper into the functions of this non-catalytic protease *in vivo*. While we observed metabolic and energy balance alterations in *Adam29*-deficient mice during phenotypical characterization, further investigations are needed to elucidate the precise role of ADAM29 in these processes. Notably, our RNA-seq analysis revealed an enrichment of genes associated with the migration of different cell types in *Adam29*-deficient liver samples, although we did not further explore the molecular mechanisms underlying ADAM29 function in this context. Additionally, while our data demonstrated the influence of this protease on the wound healing process in mice, how ADAM29 mediates this phenotype is yet to be investigated. Exploring the specific roles of ADAM29 in cell migration and its interplay with metabolic changes or the wound healing process would be a promising avenue for future research.

## STAR★Methods

### Key resources table

**Table:** 

REAGENT or RESOURCE	SOURCE	IDENTIFIER
**Chemicals, peptides, and recombinant proteins**		

EmbryoMax® Injection Buffer	Millipore (Merck)	Cat# MR-095-10F
D(+)-Glucose anhydrous	VWR Chemicals	Cat# 24379.294
Insulin	Sigma-Aldrich (Merck)	Cat# I9278-5ML
Ketamin hydrochloride (Imalgene)	Merial Laboraries	N/A
Xylazine injection (Rompun®)	Bayer	N/A
Isoflurane (IsoFlo®)	Zoetis Inc.	N/A
Formaldehyde solution	SAFC (Merck)	Cat# 1040021000
TRIzol	Invitrogen (Thermo Fisher Scientific)	Cat# 15596018
Polybrene	Santa Cruz Biotechnology	Cat# sc-134220
Lipofectamine Transfection Reagent	Invitrogen (Thermo Fisher Scientific)	Cat# 18324012
PLUS Reagent	Invitrogen (Thermo Fisher Scientific)	Cat# 11514015
Dulbecco’s Modified Eagle Medium	Gibco (Thermo Fisher Scientific)	Cat# 11965092
Fetal Bovine Serum	Gibco (Thermo Fisher Scientific)	Cat# 26140079
Penicillin-Streptomycin-L-glutamine	Gibco (Thermo Fisher Scientific)	Cat# 10378016
Antibiotic-Antimycotic	Gibco (Thermo Fisher Scientific)	Cat# 15240062
Non-Essential Amino Acids	Gibco (Thermo Fisher Scientific)	Cat# 11140035
HEPES	Gibco (Thermo Fisher Scientific)	Cat# 15630080
2-mercaptoetanol	Gibco (Thermo Fisher Scientific)	Cat# 31350010
Sodium Pyruvate	Gibco (Thermo Fisher Scientific)	Cat# 11360070
Trypsin-EDTA	Gibco (Thermo Fisher Scientific)	Cat# 2520072
DPBS (no calcium, no magnesium)	Gibco (Thermo Fisher Scientific)	Cat# 14200083

**Critical commercial assays**		

MEGAshortscript™ T7	Invitrogen (Thermo Fisher Scientific)	Cat# AM1354
MEGAclear™ kit	Invitrogen (Thermo Fisher Scientific)	Cat# AM1908
Qubit RNA HS Assay Kit	Invitrogen (Thermo Fisher Scientific)	Cat# Q10210
Mouse adiponectin ELISA kit	Millipore (Merck)	Cat# EZMADP-60K
Mouse leptin ELISA kit	Millipore (Merck)	Cat# EZML-82K
TruSeq Stranded mRNA library	Illumina	N/A
Phosphatase alkaline staining	Sigma-Aldrich (Merck)	86R-1KT

**Deposited data**		

Raw RNAseq data	This paper	ENA: PRJEB64597

**Experimental models: Cell lines**		

HEK-293T	DSMZ	ACC 635
*Adam29*^*+/+*^ and *Adam29*^*–/–*^ MEFs	This paper	N/A

**Experimental models: Organisms/strains**		

Mouse: C57/BL6	Mice bred in-house	N/A
Mouse: *Adam29*^*–/–*^ mice (C57/BL6)	This paper	N/A

**Oligonucleotides**		

Mouse *Adam29* tracRNA-sgRNA_1	IDT	**4 nmol Ultramer® DNA Oligo** AAAAAAGCACCGACTCGGTG CCACTTTTTCAAGTTGATAAC GGACTAGCCTTATTTTAACTT GCTATTTCTAGCTCTAAAACG ATCAGAGTAGGTGAACACTC CCTATAGTGAGTCGTATTA
Mouse *Adam29* tracRNA-sgRNA_2	IDT	**4 nmol Ultramer® DNA Oligo** AAAAAAGCACCGACTCGGTG CCACTTTTTCAAGTTGATAAC GGACTAGCCTTATTTTAACTT GCTATTTCTAGCTCTAAAACA GGTTATGTGGAGGGTGACTC CCTATAGTGAGTCGTATTA
T7 Promoter	Sigma-Aldrich (Merck)	TAATACGACTCACTATAGGG
*Adam29* Fwd	IDT	AAACTTGGAGGCCAGAGATACA
*Adam29* Rev	IDT	CAAAACAGGAACTGAGGGAAAC
*Adam29* Fwd 6’FAM	Sigma-Aldrich (Merck)	AAACTTGGAGGCCAGAGATACA

**Recombinant DNA**		

pMXs-Oct3/4	Addgene	Plasmid #13366
pMXs-Sox2	Addgene	Plasmid #13367
pMXs-Klf4	Addgene	Plasmid #13370

**Software and algorithms**		

Peak Scanner™ Software v1.0	Applied Biosystems (Thermo Fisher Scientific)	Cat# 4381867
FinchTV	Geospiza Inc.	https://digitalworldbiology.com/FinchTV; RRID: SCR_005584
GraphPad Prism	GraphPad (Dotmatics)	https://www.graphpad.com/; RRID: SCR_002798
ImageJ software	ImageJ	https://imagej.net/ij/; RRID: SCR_003070
Salmon	Patro et al.^43^	https://combine-lab.github.io/salmon/; RRID: SCR_017036
GSEA software	Subramanian et al.^44^ Mootha et al.^45^ Liberzon et al.^46^	https://www.gsea-msigdb.org/gsea/index.jsp; RRID: SCR_003199
IGV (Integrative Genomic Viewer)	Robinson et al.^47^	https://igv.org/; RRID: SCR_011793
STAR	Dobin et al.^48^	https://github.com/alexdobin/STAR; RRID: SCR_004463
*Wound_healing_size_tool* plugin for ImageJ software	Suarez-Arnedo et al.^49^	https://github.com/AlejandraArnedo/Wound-healing-size-tool/wiki

**Other**		

Cas9 mRNA (5meC, Psi)	Trilink	Cat# L-6125-100
High-Fat Diet	TestDiet	Cat# 58Y1
7-mm Acu-Punch	Supplier: Acuderm Inc Fisher Scientific (Thermo Fisher Scientific)	Cat# NC9410256
Culture-Insert 2 Well in 24-well black μ-Dish	Ibidi	Cat# 80242
Qubit 2.0 Fluorometer	Invitrogen (Thermo Fisher Scientific)	RRID: SCR_020553
Nanodrop Spectrophotometer	Thermo Scientific (Thermo Fisher Scientific)	RRID: SCR_018042
ABI PRISM® 3130xl Genetic Analyzer	Applied Biosystems (Thermo Fisher Scientific)	N/A
Accu-Check Aviva glucometer	Roche Diagnostics	N/A
Skyla VB1 Veterinary Clinical Chemistry Analyzer and panels	Lite-On Technology Corporation	N/A
Oxymax/CLAMS cage system	Columbus Instruments	RRID: SCR_016718
Illumina NovaSeq6000	Illumina	RRID: SCR_016387
Zeiss AxioObserver microscope	Zeiss	N/A

### Resource availability

#### Lead contact

Further information and requests for resources and reagents should be directed to and will be fulfilled by the lead contact, Diana Campos-Iglesias (diana.campos.iglesias@gmail.com) or by José M.P. Freije (jmpf@uniovi.es).

#### Materials availability

All unique reagents generated in this study are available from the lead contact in accordance with the relevant material transfer agreements.

#### Data and code availability

• RNAseq original data can be accessed at the European Nucleotide Archive (ENA) under accession ENA: PRJEB64597.
• This paper does not report original code.
• Any additional information required to reanalyze the data reported in this paper is available from the lead contact upon request.

### Experimental model and study participant details

#### Animals

Animals were housed in a pathogen-free facility under a photoperiod of 12 h light/12 h dark, 22 ± 2°C of temperature, 50 ± 10% of relative humidity and *ad libitum* access to water and food. High-fat diet (HFD) contained 60% energy from fat (TestDiet, 58Y1). *Adam29*^*+/+*^ and *Adam29*^*–/–*^ mice were maintained on a C57/BL6 background. The gender and age of the animals used in each experiment are indicated in the main text and/or their corresponding figure legend. All animal procedures were conducted in accordance with European Directive 2012/63/UE and were approved by the Committee of Animal Experimentation of the University of Oviedo and authorized by the Government of the Principality of Asturias (code: PROAE 29/2019).

#### Cell lines

Human embryonic kidney (HEK) 293T cells were maintained in 1X Dulbecco’s Modified Eagle Medium (DMEM) supplemented with 10% fetal bovine serum (FBS) at 37°C in a 5% CO_2_ incubator. In the case of *Adam29*^*+/+*^ and *Adam29*^*–/–*^ mouse embryonic fibroblasts (MEFs), 1X non-essential amino acids, 10 mM HEPES buffer, 100 μM 2-mercaptoethanol and 1X sodium pyruvate (all from Gibco) were also added to the previous medium and 15% FBS was used.

### Method details

#### Generation of *Adam29*-deficient mice

To generate *Adam29-*deficient mice, we designed two specific sgRNAs against the coding sequence of *Adam29* mouse gene (5′-AGTGTTCACCTACTCTGATC-3′ and 5′-AGTCACCCTCCACATAACCT-3′). The tracRNA-sgRNA sequences, preceded by a T7 promoter sequence were purchased as 4 nmol Ultramer® DNA Oligo (Integrated DNA Technologies). Then, *in vitro* transcription reactions were carried out for both sgRNAs with MEGAshortscript™ (Invitrogen), following manufacturer’s instructions. The resulting RNAs were purified using MEGAclear™ kit (Invitrogen) and quantified using Qubit fluorometric quantification assay (Life Technologies). The sgRNAs (50 ng/μl) and the Cas9 mRNA (100 ng/μl) (Trilink) were diluted in EmbryoMax® Injection Buffer (Merck Millipore) and microinjected into fertilized eggs from C57/BL6N mice.

#### Fragment analysis and Sanger sequencing

Genomic DNA was isolated from mouse tail biopsies using alkaline lysis buffer (NaOH 25 mM, EDTA pH = 8, 0.2 mM) followed by 99°C incubation and posterior neutralization (Tris pH = 7.4, 40 mM). We performed PCR amplification of the target region with the following primers: *Adam29* Forward 5′AAACTTGGAGGCCAGAGATACA3′ and *Adam29* Reverse 5′CAAAACAGGAACTGAGGGAAAC3′ under the following conditions: denaturation at 94°C for 30 s, annealing at 60°C for 30 s, and extension at 72°C for 30 s, 30 cycles. Forward oligonucleotide was labelled with 6FAM fluorophore in the 5′ position. The PCR products were resolved by capillary electrophoresis on an ABI PRISM® 3130xl Genetic Analyzer (Servicios Científico-Técnicos, Universidad de Oviedo) and the fragment profiles were then analyzed using Peak Scanner™ software (v1.0). Each PCR reaction was then subjected to Sanger sequencing analysis and the resulting electropherograms were visualized using FinchTV v.1.4.0.

#### Blood and plasma parameters

*Adam29*^*+/+*^ and *Adam29*^*–/–*^ mice were fasted 16 h (for glucose tolerance test) or 6 h (for insulin tolerance test). After that time, mice received intraperitoneal injection of glucose (1g per kg of body weight) or insulin (1U per kg of body weight). For blood glucose determination, blood samples were obtained from the tail vein and measured with Accu-Check Aviva glucometer (Roche Diagnostics). Areas under the curve were calculated using GraphPad Prism software. For all the other measurements, blood was extracted via cardiac puncture after anaesthetizing the mice and collected into heparinized-coated tubes. Blood was then centrifuged at 1000 x *g* at 4°C, and the supernatant was stored at -80°C until analysis. Plasma adiponectin and leptin levels were measured by using ELISA Kits from EMD Millipore (EZMADP-60K and EZML-82K, respectively), according to the manufacture’s protocol. Levels of the different biochemical parameters shown in Table 1 were determined with Diagnosis-II Panel cartridges using a Skyla VB1 Veterinary Clinical Chemistry Analyzer (Lite-On Technology Corporation, Taiwan).

#### Histological analysis

Liver sections were fixed in 4% buffered paraformaldehyde solution and embedded in paraffin by standard procedures. Paraffin sections were stained with hematoxylin and eosin (H&E) for liver tissue evaluation. NAFLD scoring system was performed as described previously.^50^

#### Indirect calorimetry

Open-circuit indirect calorimetry was performed using Oxymax/CLAMS cage system (Columbus Instruments). Mice were housed individually and kept on chow or high-fat diet on a 12:12-h light-dark cycle. Oxygen consumption (VO_2_), carbon dioxide production (VCO_2_) and movement data were recorded during 24-h period. Derivative measures (respiratory exchange ratio/RER and heat production) were calculated by integrated software. Energy expenditure (EE) was calculated as heat normalized by lean mass. Locomotor activity was measured by infrared beam breaks within the cages in the XY plane. The percent relative cumulative frequency (PRCF) was calculated as previously described.^20^

#### RNA-seq and transcriptome analysis

Total RNA of liver and testis samples was isolated using TRIzol reagent (Invitrogen), following the manufacturer’s instructions. RNA-seq libraries were prepared with the TruSeq Stranded mRNA library (Illumina). The libraries were sequenced in paired-end (2 x 150 bp) on an Illumina NovaSeq6000. Reads were quantified with *Salmon*^43^ and then imported into R to perform differential gene expression analysis with DESeq2 package (v1.40.1). Pre-rank GSEA was conducted using the GSEA software (4.1.0) and mh.all.v2023.1.Mm.symbols.gmt (Hallmark) or m5.go.bp.v2023.1.Mm.symbols.gmt (Gene Ontology Biological Process) gene set databases.^44^^,^^45^^,^^46^ Volcano and bubble plots were generated using *ggplot2* R package. For data visualization in the Integrative Genomics Viewer (IGV),^47^ reads were aligned to mm39 mouse genome using *STAR*.^48^

#### Wound healing assay

*Adam29*^*+/+*^ and *Adam29*^*–/–*^ 8-10-week-old males were anesthetized by isoflurane inhalation and the back area was shaved and disinfected with 70% ethanol. Two full-thickness excisions were made on the back of each animal with a 7-mm Acu-Punch (Acuderm), by excising the skin and *panniculus carnosus*. Healing process was monitored 11 days after injury, taking pictures of each wound 5 h later, and every day until the end of the experiment. Wound area was calculated for each wound at each time point with the ImageJ software. The mean value of both wounds was calculated for each animal and used in the subsequent graphical representations and statistical analysis.

#### Cell migration assay

*Adam29*^*+/+*^ (n = 3) and *Adam29*^*–/–*^ (n = 3) mouse embryonic fibroblasts (MEFs) were plated by triplicate on Culture-Insert 2 Well in 24-well black μ-Dish (both from Ibidi). After allowing the cells to create a monolayer, the culture-insert was removed, creating a cell-free gap of 500 μm. Then, cells were washed with 1X PBS and fresh medium was added. Cell migration was monitored under stable pressure of 5% CO_2_ in air at 37°C using a Zeiss AxioObserver microscope, at 20 min time intervals. The area of closure was measured using the *Wound_healing_size_tool* plugin for ImageJ software, as previously described.^49^

#### Mouse iPSCs generation

Mouse fibroblast reprogramming into iPSCs was performed in primary cultured cell lines of *Adam29*^*+/+*^ (n = 3) and *Adam29*^*–/–*^ (n = 3) MEFs at passage 3. Briefly, a total of four infections, supplemented with 0.8 μg/ml polybrene, were performed every 12 h with retroviral supernatants containing OCT4, SOX2, and KLF4 into MEFs seeded at 33,000 cell per well in 6-well plates. The day after the last infection, medium was replaced with fibroblast-specific medium (Dulbecco’s modified Eagle’s medium containing 15% fetal bovine serum, 1% penicillin-streptomycin-L-glutamine, 1% antibiotic-antimycotic, 1X non-essential amino acids, 10 mM HEPES buffer, 100 μM 2-mercaptoetanol, and 1X sodium pyruvate, all from Gibco). After that, medium was changed every two days, and cultures were maintained for 20 days. At that point, reprogramming efficiency was evaluated by phosphatase alkaline staining (86R-1KT, Sigma) following manufacturer’s instructions, and the number of colonies was counted using ImageJ software.

### Quantification and statistical analysis

Unless otherwise indicated, bar plots represent the mean and standard error of the mean (SEM), and the statistical significance was determined by the Student’s unpaired two-tailed *t*-test. When needed, Welch’s correction for unequal variance was applied. Data were analyzed for normal distribution using Saphiro-Wilk test. Statistical tests were performed using GraphPad Prism software. ∗p < 0.05, ∗∗p < 0.01.

## Supplemental information

Document S1. Figures S1–S4

Table S1. RNA-seq gene expression analysis using liver samples from *Adam29* wild-type and *Adam29* knock-out mice (wild-type samples set as control), related to Figure 4

Table S2. GSEA results using Hallmark as gene sets (S2A) and GO Biological Processes (S2B) as gene sets (*Adam29* knock-out vs. *Adam29* wild-type), related to Figure 4

Table S3. Analysis of *Xbp1* unprocessed and spliced transcript levels in our RNA-seq data, related to Figure 4
