# Supplementary material for: Loss of ADAM29 does not affect viability and fertility in mice but improves wound healing
Source: iScience. 2024 May 29;27(6):110135. doi: 10.1016/j.isci.2024.110135 (PMC11223086; doi:10.1016/j.isci.2024.110135)
Supplement: Document S1. Figures S1–S4 [file mmc1.pdf]

iScience, Volume *27*

Supplemental information

Loss of ADAM29 does not affect viability and fertility in mice but improves wound healing

Diana Campos-Iglesias, Alejandro A. Montero, Francisco Rodríguez, Carlos López-Otín, and José M.P. Freije

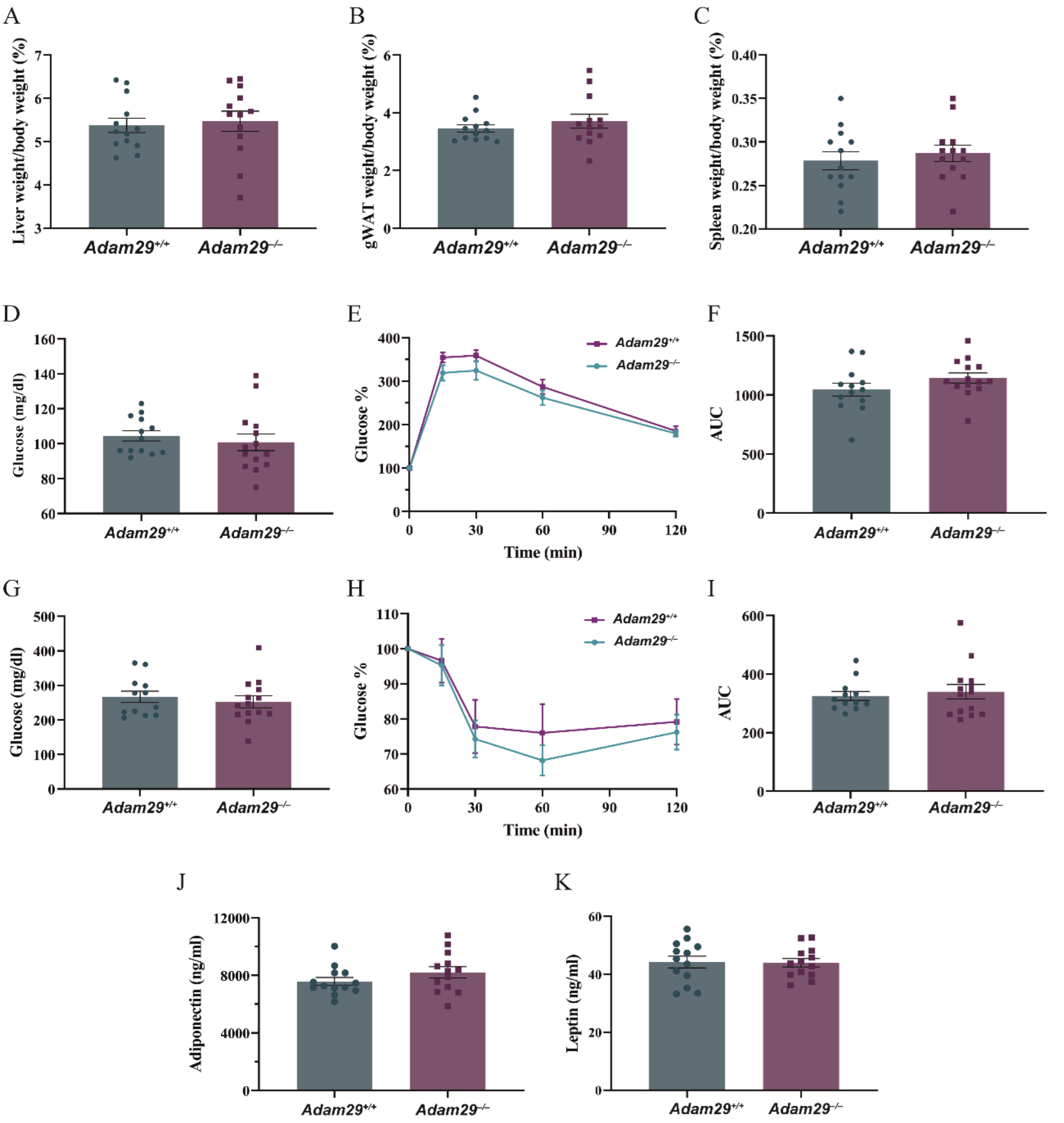

Figure S1. Effect of high fat diet (HFD) in organ weight and glucose homeostasis of *Adam29*$^{+/+}$ and *Adam29*$^{-/-}$ male mice, related to Figure 2. (**A-C**) Liver (**A**), white adipose tissue (gonadal, gWAT) (**B**), and spleen (**C**) masses were determined relative to body weight from HFD-fed *Adam29*$^{+/+}$ (n = 13) and *Adam29*$^{-/-}$ (n = 14) male mice. (**D**) Glucose levels on HFD-fed *Adam29*$^{+/+}$ and *Adam29*$^{-/-}$ mice fasted for 16 h. (**E)** Glucose tolerance test (GTT) after 16 h of fasting in HFD-fed *Adam29*$^{+/+}$ and *Adam29*$^{-/-}$ mice. The area under the curve (AUC) of the GTT is shown in (**F**). (**G**) Glucose levels on HFD-fed *Adam29*$^{+/+}$ and *Adam29*$^{-/-}$ mice fasted for 4 h. (**H**) Insulin tolerance test (ITT) after 4 h of fasting in HFD-fed *Adam29*$^{+/+}$ and *Adam29*$^{-/-}$ mice. (**I**) Area under the curve (AUC) of the ITT. (**J-K**) Plasma levels of adiponectin (**J**) and leptin (**K**) in HFD-fed *Adam29*$^{+/+}$ and *Adam29*$^{-/-}$ mice. Data are presented as mean ± SEM.

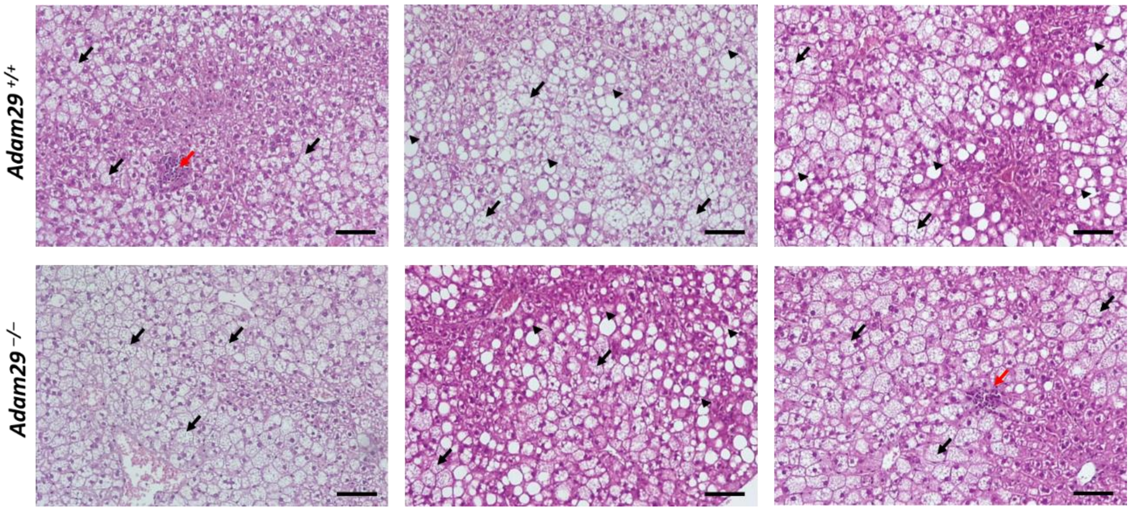

Figure S2. Liver histology of *Adam29*$^{+/+}$ and *Adam29*$^{-/-}$ HFD-fed male mice, related to Figure 2. Representative hematoxylin and eosin stained slides used for assessing NASH are depicted. Small fat droplets in hepatocytes (microvacuolar steatosis) are indicated by black arrows, while large fat droplets (microvacuolar steatosis) are indicated by arrowheads. Red arrows indicate inflammatory cell clusters. Scale bars = 100 µm.

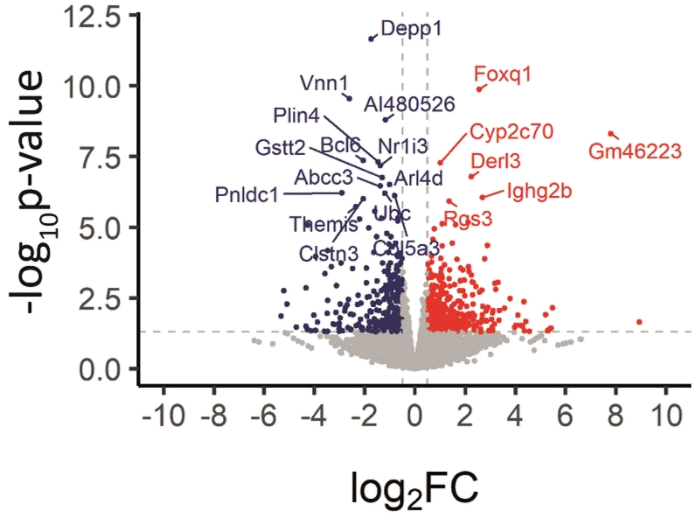

Figure S3. RNAseq analysis reveals changes in gene expression between *Adam29*$^{+/+}$ and *Adam29*$^{–/–}$ mice, related to Figure 4. Volcano plot showing -log$_{10}$ (p-value) versus log$_2$ fold change (log$_2$FC) of differentially expressed genes between *Adam29*$^{+/+}$ and *Adam29*$^{–/–}$ liver samples. Each dot represents a single gene. Red and blue dotes denote significant upregulated and downregulated genes, respectively (p-value < 0.05). The 20 most altered genes are named.

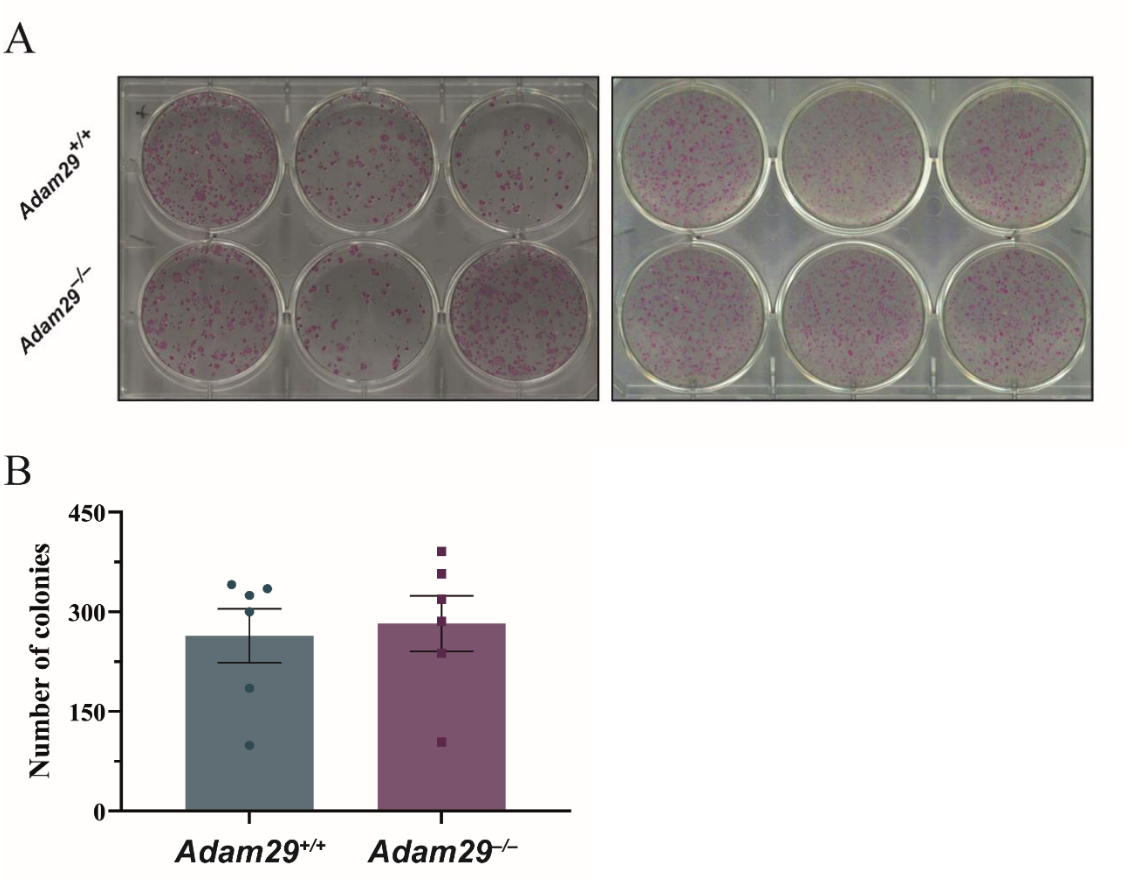

Figure S4. ADAM29 depletion does not affect reprogramming of MEFs, related to Figure 5. (**A**) Phosphatase alkaline staining of *Adam29*$^{+/+}$ and *Adam29*$^{-/-}$ iPSCs. (**B**) Number of positive colonies at the end of the experiment. Six biological replicated of each genotype were used to perform the experiment. Each point represents the mean value of three technical replicates for each cell line.
